# New Data on the *In Vitro* Activity of Fenticonazole against Fluconazole-Resistant *Candida* Species

**DOI:** 10.1128/AAC.01459-20

**Published:** 2020-11-17

**Authors:** Margherita Cacaci, Giulia Menchinelli, Riccardo Torelli, Dominique Sanglard, Maurizio Sanguinetti, Brunella Posteraro

**Affiliations:** a Dipartimento di Scienze Biotecnologiche di Base, Cliniche Intensivologiche e Perioperatorie, Università Cattolica del Sacro Cuore, Rome, Italy; b Dipartimento di Scienze di Laboratorio e Infettivologiche, Fondazione Policlinico Universitario A. Gemelli IRCCS, Rome, Italy; c Institute of Microbiology, University of Lausanne and University Hospital, Lausanne, Switzerland; d Dipartimento di Scienze Gastroenterologiche, Endocrino-Metaboliche e Nefro-Urologiche, Fondazione Policlinico Universitario A. Gemelli IRCCS, Rome, Italy

**Keywords:** *Candida*, fenticonazole, antifungal resistance

## LETTER

Despite the advent of echinocandins and newer triazoles (voriconazole, posaconazole, and isavuconazole) (1), fluconazole (FLZ) remains an important component of today’s antifungal arsenal, particularly for treatment of *Candida* infections (2, 3). However, FLZ may not be effective against *Candida* species, including C. albicans and (more frequently) C. glabrata, in cases of azole-resistant isolates (4). Except in C. krusei (a *Candida* species that is intrinsically FLZ resistant), the general and long-term therapeutic use of FLZ (or other triazoles) can result in acquisition of molecular mechanisms that enable *Candida* isolates to exhibit antifungal resistance (5). It is known that increased drug efflux pump activities result in low intracellular azole accumulation, while mutations in the 14-α-lanosterol demethylase—the primary fungal target—prevent azoles from enzyme binding (6). Fenticonazole (FEZ) is an imidazole-derived antifungal compound that, unlike triazoles, displays *in vitro* antimicrobial activity not only directed against fungal isolates (7). Thus, a peculiar FEZ mechanism of action—perhaps related to its oxidative cytotoxicity—could allow the drug not only to cure mixed fungal and bacterial infections (8) but also to overcome the main ways in which *Candida* species may acquire antifungal resistance (6).

We tested the activity of FEZ against paired isolates (i.e., parental and derivative isolates) from C. albicans (20 isolates) and C. glabrata (30 isolates) species, respectively. In each isolate’s pair, the FLZ-resistant (derivative) isolate originated from the FLZ-susceptible or susceptible-dose-dependent (parental) isolate following resistance development during patient infection (9). All except four (from bloodstream infection) isolates were from mucosal surface (e.g., oropharyngeal, vaginal, etc.) infections. All 25 FLZ-resistant (10 C. albicans and 15 C. glabrata) isolates exhibit known molecular resistance mechanisms, which consisted of upregulation of drug efflux pump-encoding genes (*CDR1*/*CDR2*, *MDR1* [only for C. albicans], and *SNQ2* [only for C. glabrata]) and/or point mutations of 14-α-lanosterol demethylase-encoding *ERG11* gene (Table S1 in the supplemental material). MIC values to FEZ and FLZ—both obtained as standard powders from Sigma-Aldrich (Milan, Italy)—were determined using the protocol specified in the Clinical and Laboratory Standards Institute (CLSI) M27-A3 document without modifications (10). Only for FLZ, MIC values were interpreted according to species (C. albicans or C. glabrata)-specific clinical breakpoints reported in the CLSI M27-S4 document (11). We used MIC values (Table S1) to calculate geometric mean (GM) MICs with 95% confidence intervals (CIs) and MIC ranges for both FEZ and FLZ antifungal drugs. We assessed statistically significant (*P* < 0.05) differences between GM MIC values obtained for isolate groups from each species (see below), using repeated-measures analysis of variance (ANOVA) on log_2_ MICs followed by Bonferroni-Dunn’s multiple-comparison test (12).

**FIG 1:**
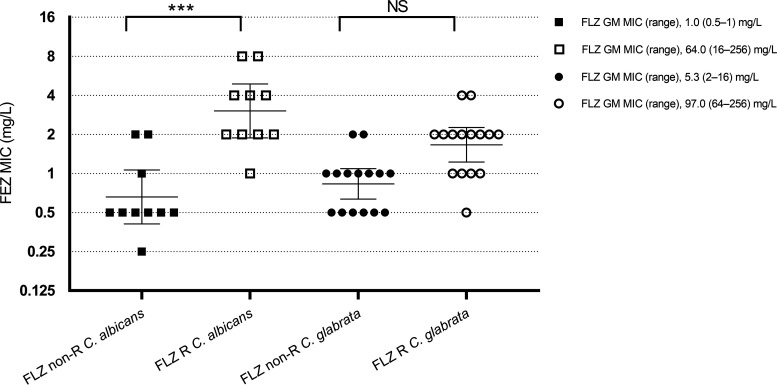
Distribution of FEZ MICs for clinical C. albicans and C. glabrata isolates without (nonresistant [non-R] isolates) or with (resistant [R] isolates) molecular mechanisms contributing to FLZ resistance phenotype. Shown is the presence or absence of statistical significance (*P* < 0.001; NS, no significance) between the FEZ GMs of isolate groups represented by a horizontal line within each plot, which displays individual FEZ MIC points. Error bars indicate confidence intervals.

Of 25 isolates with molecular mechanisms contributing to the FLZ resistance phenotype observed *in vitro*, 15 C. glabrata and 1 C. albicans isolate overexpressed drug efflux pumps alone, whereas 9 C. albicans isolates combined overexpression of drug efflux pumps and *ERG11* amino acid substitution(s) (Table S1). For C. albicans isolates, FEZ MIC ranges were 0.25 to 2 mg/liter among FLZ-nonresistant isolates (MICs, 0.125 to 1 mg/liter) and 1 to 8 mg/liter among FLZ-resistant isolates (MICs, 16 to 256 mg/liter). For C. glabrata isolates, FEZ MIC ranges were 0.5 to 2 mg/liter among FLZ-nonresistant isolates (MICs, 2 to 16 mg/liter) and 0.5 to 4 mg/liter among FLZ-resistant isolates (MICs, 64 to 256 mg/liter). Figure 1 shows the distribution of FEZ MICs in the FLZ-nonresistant or resistant isolates of C. albicans and C. glabrata, respectively. Interestingly, the GM MICs ± CIs of C. albicans FEZ MICs in FLZ-nonresistant isolates differed significantly from that in FLZ-resistant isolates (GM MIC of 0.65 [95% CI, 0.40 to 1.06] versus GM MIC of 3.03 [95% CI, 1.87 to 4.89]; *P* < 0.001). Conversely, no significant difference was seen between the GM ± CIs of C. glabrata FEZ MICs in FLZ-nonresistant isolates and that in FLZ-resistant isolates (GM MIC of 0.83 [95% CI, 0.63 to 1.08] versus GM MIC of 1.66 [95% CI, 1.22 to 2.25]; *P = *0.26).

In conclusion, we showed that FEZ MIC values were lower than FLZ MIC values in 50 well-characterized isolates from two clinically relevant *Candida* species, including C. albicans and C. glabrata. Remarkably, differences were more prominent in FLZ-resistant isolates than their nonresistant counterparts but were statistically significant only for C. albicans. Our data demonstrate that FEZ exhibits higher activity than FLZ. FEZ activity was less dependent on drug efflux pump-mediated FLZ resistance in *Candida* species such as C. glabrata. Based on these findings, FEZ should be evaluated as a candidiasis treatment, particularly in patients with recurrent or antifungal-recalcitrant *Candida* infections.

## Supplementary Material

Supplemental file 1
