# Supplementary material for: New Data on the In Vitro Activity of Fenticonazole against Fluconazole-Resistant Candida Species
Source: Antimicrob Agents Chemother. 2020 Nov 17;64(12):e01459-20. doi: 10.1128/AAC.01459-20 (PMC7674059; doi:10.1128/AAC.01459-20)
Supplement: Supplemental file 1 [file AAC.01459-20-s0001.pdf]

TABLE S1 Detailed antifungal susceptibility characterization of 50 paired *Candida* clinical isolates included in the study[a]

Isolate designation	Isolate type	MIC (mg/liter)[b]		FLZ susceptibility category[c]	Efflux pump gene(s) overexpression (fold increase)[d]	ERG11 amino acid substitution[e]
		FEZ	FLZ			
C. albicans isolates						
DSY281	Parental	2	1	S	None	None
DSY284	Derivative	4	256	R	*CDR1* (25.2), *CDR2* (30.3)	S405F
DSY290	Parental	0.5	0.25	S	None	None
DSY292	Derivative	1	64	R	*CDR1* (61.1), *CDR2* (12.4)	G464S, R467K, Y132H
DSY294	Parental	0.5	0.25	S	None	None
DSY296	Derivative	4	64	R	*CDR1* (15.5), *CDR2* (16.3)	G464S
DSY2321	Parental	0.25	0.5	S	None	S405F
DSY2322	Derivative	2	32	R	*CDR2* (25.2), *MDR1* (8.5)	S405F
DSY544	Parental	2	0.125	S	None	None
DSY775	Derivative	8	128	R	*CDR1* (11.4), *CDR2* (26.3)	G464S
DSY2243	Parental	1	1	S	None	S442F, R467K
DSY2242	Derivative	2	16	R	*CDR1* (5.7), *CDR2* (7.1)	S442F, R467K
DSY550	Parental	0.5	0.25	S	None	None
DSY551	Derivative	8	256	R	*CDR2* (2.3)	G464S, Y132H
DSY741	Parental	0.5	0.5	S	None	None
DSY742	Derivative	2	16	R	*MDR1* (1024.1)	None
DSY757	Parental	0.5	0.5	S	None	None
DSY758	Derivative	2	64	R	*CDR1* (12.1), *CDR2* (14.9)	G464S, F145L
DSY2250	Parental	0.5	1	S	None	S442F, G464S
DSY2251	Derivative	4	64	R	*CDR1* (39.4), *CDR2* (10.8), *MDR1* (23.7)	S442F, G464S
C. glabrata isolates						
BPY40	Parental	1	4	SDD	None	None
BPY41	Derivative	2	256	R	*CDR1*(483.1), *CDR2* (70.3), *SNQ2* (11.7)	None
BPY112	Parental	1	2	SDD	None	None
BPY126	Derivative	1	128	R	*CDR1* (56.2), *CDR2* (5.6), *SNQ2* (18.7)	None
DSY738	Parental	0.5	16	SDD	None	None
DSY739	Derivative	2	64	R	*CDR1* (97.1), *CDR2* (2.8), *SNQ2* (2.0)	None
DSY2253	Parental	1	8	SDD	None	None
DSY2254	Derivative	2	128	R	*CDR1* (56.2), *CDR2* (5.5), *SNQ2* (18.7)	None
DSY2270	Parental	1	8	SDD	None	None
DSY2271	Derivative	1	64	R	*CDR2* (3.8)	None
DSY2276	Parental	0.5	8	SDD	None	None
DSY2277	Derivative	2	64	R	*CDR1* (3.8), *SNQ2* (8.5)	None
DSY529	Parental	0.5	4	SDD	None	None
DSY530	Derivative	2	64	R	*CDR1* (31.1), *CDR2* (3.5)	None
DSY753	Parental	1	4	SDD	None	None
DSY754	Derivative	2	64	R	*CDR1* (16.9)	None
DSY755	Parental	0.5	4	SDD	None	None
DSY756	Derivative	4	128	R	*CDR1* (173.6), *CDR2* (71.9), *SNQ2* (4.4)	None
DSY773	Parental	0.5	16	SDD	None	None
DSY774	Derivative	4	64	R	*CDR1* (18.6), *CDR2* (9.3)	None
DSY2317	Parental	2	4	SDD	None	None
DSY717	Derivative	4	128	R	*CDR1* (203.3), *SNQ2* (2.2)	None
DSY2324	Parental	1	4	SDD	None	None
DSY2325	Derivative	0.5	128	R	*CDR1* (453.8), *CDR2* (19.6), *SNQ2* (4.7)	None
DSY562	Parental	0.5	4	SDD	None	None
DSY565	Derivative	1	64	R	*CDR1* (53.1)	None
DSY1166	Parental	1	4	SDD	None	None
DSY1169	Derivative	2	128	R	*CDR1* (70.0), *CDR2* (60.9)	None
DSY1176	Parental	2	4	SDD	None	None
DSY1185	Derivative	4	128	R	*CDR1* (457.1), *CDR2* (45.3)	None

[a]Paired isolates consisted of both parental and derivative (i.e., with acquired resistance mechanism) isolates consecutively obtained from the same patient during infection. For 46 isolates, clinical samples were from mucosal surfaces such as oropharynx, vagina, or bladder samples; the remaining four isolates were from blood samples. See reference 1 and therein references for further details.

[b]For each isolate, minimum inhibitory concentration (MIC) values to fenticonazole (FEZ) and fluconazole (FLZ), respectively, were determined by the Clinical and Laboratory Standards Institute (CLSI) M27-A3 method and were expressed as mg/liter.

[c]For *C. albicans*, isolates were categorized as FLZ-susceptible (S) or -resistant (R) according to the clinical breakpoints (S, MIC ≤2 mg/liter; R, MIC ≥64 mg/liter) reported in the CLSI M27-S4 document (2). For *C. glabrata*, isolates were categorized as FLZ-SDD or -R according to the clinical breakpoints (SDD, MIC ≤32 mg/liter; R, MIC ≥64 mg/liter) reported in the CLSI M27-S4 document (2).

[d]For each derivative (FLZ-R) isolate, significant relative increases (≥2-fold) in the efflux pump gene(s) expression were determined with respect to the parental (FLZ-S or FLZ-SDD) isolate, using quantitative real-time RT-PCR based analysis as previously described (1).

[e]For each isolate, *ERG11* gene sequencing was performed as previously described (1). The aminoacid substitutions in the *ERG11* encoded protein are shown. We considered only the aminoacid substitutions associated with FLZ-R phenotypes as contributors to the isolates' FLZ resistance. Three isolates with FLZ-S phenotypes had an ERG11 aminoacid substitution, namely the isolates DSY2321 (S405F), DSY2243 (S442F, R467K), and DSY2250 (S442F, G464S).

REFERENCES

1. Posteraro B, Martucci R, La Sorda M, Fiori B, Sanglard D, De Carolis E, Florio AR, Fadda G, Sanguinetti M. 2009. Reliability of the Vitek 2 yeast susceptibility test for detection of in vitro resistance to fluconazole and voriconazole in clinical isolates of Candida albicans and Candida glabrata. J Clin Microbiol 47:1927−1930. https://doi.org/10.1128/JCM.02070-08.
2. Clinical and Laboratory Standards Institute. 2012. Reference method for broth dilution antifungal susceptibility testing of yeasts. CLSI supplement M27-S4. Clinical and Laboratory Standards Institute. Wayne, PA.
